# Adrenocortical carcinoma: presentation and outcome of a contemporary patient series

**DOI:** 10.1007/s12020-019-01918-9

**Published:** 2019-04-12

**Authors:** Iiro Kostiainen, Liisa Hakaste, Pekka Kejo, Helka Parviainen, Tiina Laine, Eliisa Löyttyniemi, Mirkka Pennanen, Johanna Arola, Caj Haglund, Ilkka Heiskanen, Camilla Schalin-Jäntti

**Affiliations:** 1 Endocrinology, Abdominal Center, Helsinki University Hospital and University of Helsinki, Helsinki, Finland; 2 Department of Surgery, Helsinki University Hospital and University of Helsinki, Helsinki, Finland; 3 HUS Medical Imaging Centre, Radiology, Helsinki University Hospital and University of Helsinki, Helsinki, Finland; 4 Children’s Hospital, Helsinki University Hospital and University of Helsinki, Helsinki, Finland; 5 Department of Biostatistics, University of Turku and Turku University Hospital, Turku, Finland; 6 Department of Pathology, University of Helsinki and HUSLAB, Helsinki, Finland

**Keywords:** Adrenocortical carcinoma, ENSAT stage, Hounsfield units, Mitotane, Surgery, Complications, Survival

## Abstract

**Background:**

Adrenocortical carcinoma (ACC) is a rare endocrine carcinoma with poor 5-year survival rates of < 40%. According to the literature, ACC is rarely an incidental imaging finding. However, presentation, treatment and outcome may differ in modern series.

**Design and methods:**

We studied all patients (*n* = 47, four children) from a single centre during years 2002–2018. We re-evaluated radiologic and histopathological findings and assessed treatments and outcome. We searched for possible *TP53* gene defects and assessed nationwide incidence of ACC.

**Results:**

In adults, incidental radiologic finding led to diagnosis in 79% at median age of 61 years. ENSAT stage I, II, III and IV was 19%, 40%, 19% and 21%, respectively. Nonenhanced CT demonstrated > 20 Hounsfield Units (HU) for all tumours (median 34 (21–45)), median size 92 mm (20–196), Ki67 17% (1–40%), Weiss score 7 (4–9) and Helsinki score 24 (4–48). ACC was more often found in the left than the right adrenal (*p* < 0.05). One child had Beckwith-Wiedemann and one a *TP53* mutation. In adults, the primary tumour was resected in 88 and 79% received adjuvant mitotane therapy. Median hospital stay was significantly shorter in the laparoscopic vs. open surgery group (4 (3–7) vs. 8 (5–38) days, respectively; *p* < 0.001). In 3/4 patients, prolonged remission of > 5 to > 10 years was achieved after repeated surgery of metastases. Overall 5-year survival was 67%, and 96% vs. 26% for ENSAT stage I–II vs. III–IV (*p* < 0.0001). ENSAT stage and Ki67 predicted survival, type of surgery did not. Mitotane associated with better survival.

**Conclusions:**

Contemporary ACC predominantly presents as an incidental imaging finding, characterised by HU > 20 on nonenhanced CT but variable tumour size (20–196 mm). Malignancy cannot be ruled out by small tumour size only. The 5-year survival of 96% in ENSAT stage I–III compares favourably to previous studies.

## Introduction

Adrenocortical carcinoma (ACC) is a rare and aggressive endocrine malignancy that can occur at any age. In adults, the estimated incidence of ACC is one per million per year [1, 2]. Most series report 5-year survivals < 40% [1, 3–11]. In children < 3 years, 5-year disease-free survival after total resection is 80%, while in those > 13 years survival is 40%, similar to that in adults [2, 12]. Disease stage, best defined by the European Network for the Study of Adrenal Tumours (ENSAT stage), radical surgery, age, Ki67 and Helsinki Score seem to predict survival [2, 3, 13–15]. Adjuvant mitotane is recommended in adult patients with high risk of recurrence [16, 17] and mitotane monotherapy or combined mitotane and etoposide-doxorubicin cisplatin treatment for advanced disease [18, 19].

Previously, 10–15% of ACCs have presented as incidental imaging findings [20, 21], while reported prevalence of ACC among adrenal incidentalomas has varied between 1–11% [22]. However, incidentally discovered asymptomatic adrenal masses are getting more common due to increasing use of imaging [22, 23]. We hypothesised that presentation and outcome of ACC may differ in modern compared to old series. We here report data on presentation, treatment and outcome in a contemporary series, including all patients from a single centre diagnosed in 2002 to 2018, and nationwide incidence of ACC in 2001–2015.

## Subjects and methods

### Patient population

Patients were identified from the Helsinki University Hospital pathology registry and electronic patient records using ICD-10 codes C74.0 and 74.9. All cases (Weiss score ≥ 4 [24, 25]) diagnosed between 1 January 2002 and 27 April 2018 were included.

### Histopathological evaluation

Two endocrine pathologists (M.P., J.A.) re-evaluated all tumours and determined Ki67, Weiss and Helsinki scores [13]. One tumour with Weiss score 3 was excluded.

### Radiologic evaluation

Preoperative computed tomography (CT) images were re-evaluated with the Agfa Impax PACS software by an abdominal radiology specialist (H.P.). Tumour size was defined as largest axial plane diameter. Presence or absence of tumour thrombus or distant metastases was evaluated from contrast-enhanced images. Hounsfield units (HU) were measured from noncontrast images by placing a single round region of interest (ROI) on the tumour. The ROI covered the largest possible part of the tumour plane, avoiding necrosis, haemorrhage and calcifications.

### Data collection

Data on clinical presentation, laboratory measurements, tumour characteristics, surgery, histopathology and adjuvant treatments during years 2002 to 2018 were collected from our electronic patient records. All laboratory tests, including germline gene mutation testing, were performed with in house methods in the Helsinki University Hospital Central Laboratory, HUSLAB. We used ENSAT Criteria (stage I = tumour diameter ≤ 5 cm, stage II tumour diameter > 5 cm, stage III = infiltration of neighbouring structures, venous tumour thrombosis in caval or renal vein, or positive lymph nodes, stage IV = distant metastases) [3]. We registered recurrences and deaths. We retrieved nationwide number of ACC cases and age standardised 5-year incidences from the Finnish Cancer Registry; www.cancersociety.fi.

The study protocol was approved by the board review of the Abdominal Centre, Helsinki University Hospital.

### Statistical analysis

Data is presented as median (interquartile range, IQR, or range) for continuous variables. For categorical data, rates and proportions were calculated. Fisher exact test was used for calculation of differences between groups. Mean ranks between groups were compared with Mann-Whitney-*U*-test. Median survival was calculated according to Kaplan–Meier and predictors of survival with Cox proportional hazards regression analyses. All reported *p*-values are two-sided. *p*-values < 0.05 (two-tailed) were considered statistically significant. Ki-67 was grouped in three categories: (1) 1–9%, (2) 10–19% and (3) ≥ 20%. Calculations were performed with SPSS25.

## Results

### Patient characteristics

Altogether 47 patients, 4 (9%) being children, were diagnosed with ACC. In adults, median age at diagnosis was 61 years (IQR 53–66), 25 (58%) were female and median follow-up was 35 months (IQR 8–89). At presentation, 15/42 (36%) had non-specific abdominal pain and 6/42 (14%) bruises; 5/25 (24%) women had hirsutism and the youngest woman (20 years) menstrual disturbances. Eight patients had potassium concentrations < 3.5 mmol/l ( < 3.0 mmol/l in 7, lowest 1.6 mmol/l). Sixty-three percent had biochemically verified adrenal hormone excess. Isolated hypercortisolism (41%) was most common, followed by excess co-secretion of cortisol and androgens (22%), isolated hyperandrogenism (19%), co-secretion of aldosterone and cortisol and aldosterone (11%) and isolated aldosterone excess (7%).

### Characteristics of children with adrenocortical carcinoma

**Fig. 1:**
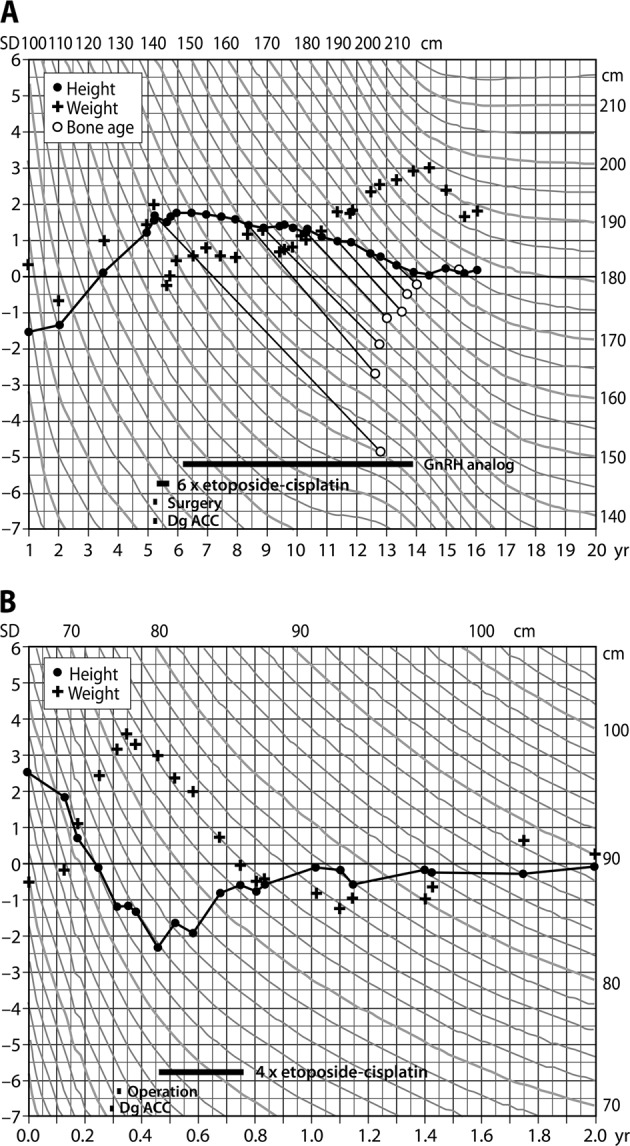
**a** Growth chart demonstrates accelerated growth in a 5-year-old boy with androgen secreting ACC. Hyperandrogenism resulted in advanced bone age (12.8 years) and subsequent precocious central puberty that was treated with GnRH-analogue therapy from 6.2 to 13.8 years of age. **b** Growth chart of a 0.3-year-old boy with cortisol secreting ACC causing weight gain and decreased height velocity

Characteristics of children with ACC are given in Table 1. Patients 3 and 4 presented with clinical signs of hyperandrogenism, and patient 1 with signs of hypercortisolism. Figure 1 demonstrates typical growth charts in hyperandrogenism (patient 4; Fig. 1a) and hypercortisolism (patient 1, Fig. 1b). Patient 2 was asymptomatic with normal biochemistry, and diagnosed with an adrenal tumour when undergoing abdominal ultrasound screening because of familial Li-Fraumeni syndrome (Table 1).

**Table 1:** Characteristics of paediatric patients (*n* = 4) with ACC

	Patient #1	Patient #2	Patient #3	Patient #4
Age (years)	0.3	1.3	1.6	5.2
Sex	Male	Female	Female	Male
Symptoms and signs	Developmental delay, hypertension, increased weight, decreased length growth velocity, left ventricle hypertrophy	None	Pubic hair, clitoromegaly	Pubic hair, penile growth, increase in height, deepening of voice, advanced bone age of 12.8 years
Biochemically verified hormone excess	Hypercortisolism	None	Hyperandrogenism	Hyperandrogenism
Tumour size (mm)	59	24	57	33
Location	Right adrenal	Right adrenal	Right adrenal	Left adrenal
Weiss score	5	7	6	6
ENSAT stage	II	I	II	I
Genetics	Hypomethylation of *KCNQ1OT* gene (Beckwith-Wiedemann syndrome)	Germline *TP53* mutation (Li-Fraumeni syndrome)	None	None

Time between diagnosis and surgery was 7, 8 and 16 days in symptomatic patients and 51 days for the nonsymptomatic child. Three children were discharged on the fourth postoperative day, postoperative recovery of patient 1 included 2 days in ICU, 49 days in the paediatric ward and chemotherapy on the 52nd postoperative day. All children received 4–6 cycles of adjuvant etoposide-cisplatin therapy. At 7.9 years (1.5–11.8 years) of follow-up, all children are in remission and have reached the developmental milestones according to age. So far, children with Beckwith-Wiedemann and Li-Fraumeni syndrome have not displayed other manifestations. Patient 4 predictably developed precocious central puberty and received GnRH-analogue therapy from 6.2 to 13.8 years of age (Fig. 1a). After cessation of GnRH-therapy, puberty proceeded normally and he reached his adult target height.

### TP53 and other gene defects and cancers in patients with adrenocortical carcinoma

Gene defects in children are presented in Table 1. Ten adult patients (age < 20 years (*n* = 3), concomitant other cancers (follicular lymphoma, earlier ACC and breast cancer, lung adenocarcinoma, prostate cancer (*n* = 4)) or strong family history of cancer (*n* = 3)) were screened for germline *TP53* gene mutations, all were negative.

### Nationwide incidence of adrenocortical carcinoma during years 2006–2015

The nationwide number of new cases was 32 during years 2006–2010, and 41 during 2011–2015, respectively. The corresponding 5-year age standardised incidence rates per one million person-years were 0.9 and 1.0 in years 2006–2010 and 2011–2015, respectively.

### Radiologic characteristics of adrenocortical carcinoma and ENSAT staging in adult patients

Median tumour size was 91.5 mm (IQR 52.3–133.0) with no significant gender difference (*p* = 0.98) (Table 2). Thirty-one (72%) tumours originated from the left and 12 (28%) from the right adrenal (*p* = 0.046). Thirty-four (79%) of the tumours were incidental imaging findings. Median tumour density (30/43 patients) was 33.5 HU (IQR 28.5–38). HU was equally high in men and women (32.5, IQR 25.5–36.5 vs 34.0, IQR 29.4–39.5, respectively, *p* = 0.31) On imaging, one patient had two tumour masses (both HU > 21) in the same adrenal.

**Table 2:** Radiologic and histologic characteristics of the tumours

Variable	Median	Range
Tumour size (mm)^a^	91.5	20–196
Radiopacity (HU)	33.5	21–45
Ki67 (%)	17	1–40
Weiss score	7	4–9
Helsinki score	25	4–48
	n	%
ENSAT stage^a^		
I	8	19
II	17	40
III	8	19
IV	9	21
Lateralisation		
Left adrenal	31	72**
Right adrenal	12	28

**p = 0.046 assuming equal distribution

^a^For one patient, size and stage at presentation are not known

At presentation, 8 patients (19.0%) had stage I disease, 17 (40.5%) stage II, 8 (19.0%) stage III and 9 (21.4%) stage IV (Table 2). There was no significant difference in the proportion of men and women in ENSAT groups I–II compared to III–IV (*p* = 1.0).

### Surgery in adult patients with adrenocortical carcinoma

Surgical characteristics are given in Table 3. The primary tumour was operated in 38 (88%) patients. One tumour was removed after prolonged pre-treatment with mitotane. One patient was attempted to operate on twice, but was inoperable due to vascular tumour involvement. Four patients were not operated on (two with large metastatic tumour burden, two with significant surgical risks). Twenty surgeries were open adrenalectomies, 16 laparoscopic and 3 other laparoscopic surgeries were converted to open surgery (Table 3). The laparoscopic group included successful surgery of four tumours ranging from 60–100 mm. Additional organs (eight kidneys, five spleens, three partial resections of pancreas, one partial liver resection) were resected in ten operations.

**Table 3:** Surgical features and outcomes

	All adult patients (*n* = 43)	Converted (*n* = 3) adrenalectomies	Open (*n* = 20)^a^ and converted (*n* = 3)^b^ adrenalectomies (*n* = 23)	Laparoscopic adrenalectomies (*n* = 16)^c^	*p*-value (comparison between laparoscopic and open surgery)
Patient characteristics					
Age	61 (18–84)	63 (63–83)	63 (18–84)	55.5 (20–69)	0.039
Female sex	25 (58%)	2 (67%)	13 (52%)	11 (69%)	0.51
Tumour characteristics					
Axial size (mm)	91.5 (20–196)	74 (69–76)	122 (69–196)	44 (20–96)	<0.0001
<60 mm	12 (29%)	0 (0%)	0 (0%)	11 (73%)	
60–100 mm	11 (26%)	3 (100%)	6 (26%)	4 (27%)	
>100 mm	19 (45%)	0 (0%)	17 (74%)	0 (0%)	
ENSAT stage					0.002^d^
I	8 (19%)	0 (0%)	0 (0%)	8 (53%)	
II	17 (40%)	2 (67%)	12 (52%)	5 (33%)	
III	8 (19%)	1 (33%)	5 (22%)	1 (6%)	
IV	9 (21%)	0 (0%)	6 (26%)	1 (6%)	
Lateralisation					
Left	31 (72%)	3 (100%)	18 (78%)	10 (63%)	0.47
Right	12 (28%)	0 (0%)	5 (22%)	6 (37%)	
Complications^e^	9	1 (33%)	8 (35%)	1 (6%)	0.11
Grade I	2	1 (33%)	2 (9%)	0 (0%)	
Grade II	3	0 (0%)	3 (13%)	0 (0%)	
Grade III	3	0 (0%)	2 (9%)	1 (6%)	
Grade IV	1	0 (0%)	1 (4%)	0 (0%)	
Rupture of tumour capsule	4	0 (0%)	3 (13%)	1 (6%)	1.0
Length of hospitalisation	6 (3–38)	8 (6–9)	8 (5–38)	4 (3–7)	<0.0001
Follow-up (days)	1325 (22–7638)	260 (36–961)	1082 (22–5530)	1492.5 (576–7638)	0.23

Comparison of open and laparoscopic surgery subgroups

Characteristics of the 39 patients who underwent surgery, including one patient that was inoperable ^a^despite surgical attempts

^b^Cause of conversion: (6 cm and 9 cm); view obstructed by tumour, (8 cm); tumour adherent to pancreas. Continuous variables are presented as medians and range

^c^For a single patient, tumour size and ENSAT stage is not known

^d^Comparing ENSAT I–II to ENSAT III–IV

^e^Graded according to Clavien-Dindo classification of surgical complications [26]

Surgery was significantly more common in ENSAT stage I–II compared to III–IV (25/25 (100%) vs. 12/17 (71%), respectively, *p* = 0.007), and open surgery (laparotomy or conversion) more common than laparoscopic surgery in ENSAT III–IV compared to I–II (*p* = 0.002). Median tumour size was smaller in the laparoscopic compared to the open surgery group (Table 3, *p* < 0.0001). Rupture of tumour capsule occurred in four surgeries, and was unrelated to the surgical method (*p* = 1.0).

There was no surgical mortality, but seven (16%) patients had postoperative complications, graded according to Clavien-Dindo [26] (Table 3). Infections were most common (sepsis (*n* = 2), abscess (*n* = 2), pneumonia (*n* = 2), wound infection (*n* = 2), infection of uncertain origin (*n* = 1)). Injury of diaphragm caused herniation of the gastric fundus in one patient, requiring surgical correction. The incidence of complications was unrelated to the surgical method (open surgery vs. laparoscopy, *p* = 0.11). Median hospital stay was significantly longer in the open compared to laparoscopic surgery group (median 8 compared to 4 days, respectively; *p* < 0.0001).

### Surgery in metastatic disease

Metastases were resected in four patients (lung metastases, lung metastases followed by three additional resections of lung metastases, lung metastases followed by resection of liver metastases, resection of lymph node metastasis). Two of these patients have achieved sustained remission of > 5 years, and another patient is alive with > 10 years survival and unremarkable CT in November 2018.

### Histopathology

Median Weiss score did not differ between men and women (6.0 (IQR 5.0–8.0) vs. 7.0 (IQR 6.0–9.0), respectively; *p* = 0.24), neither did Helsinki score (20.0, IQR 10.3–31.8 vs. 28, IQR 15.0–36.5, *p* = 0.42) or Ki-67 (11.0%, IQR 5.5–23.8 vs. 20%, IQR 10.0–30.0, *p* = 0.22),.

### Systemic treatments

Data on systemic treatments is given in Table 4. Thirty-four (79%) patients received adjuvant mitotane therapy. Twenty-two of them belonged to ENSAT stage I–II and 11 to III–IV. All except one fulfilled the indications for mitotane treatment as suggested by Fassnacht et al. [27]. Administration of mitotane therapy was not related to ENSAT stage (*p* = 0.12). Target concentrations were reached in 18 (60%) patients in 250 days (78–1055) with a cumulative dose of 870 g (169–2334). Median duration of mitotane therapy was 896 days (37–2786). Side-effects were common (Table 5) and treatment was discontinued in 11 (32%) patients because of liver toxicity, gastrointestinal and neurocognitive side-effects (Table 5).

**Table 4:** Treatment modalities used for ACC

Treatment modality	*n*	%
Surgical resection alone	6	14
Surgical resection and mitotane	24	56
Surgical resection, mitotane and other systemic treatment	8	19
Mitotane and other systemic treatment	2	5
Systemic treatment other than mitotane	2	5
Only palliative treatment	1	2

**Table 5:** Mitotane-associated adverse effects and reasons for discontinuation

Adverse effects	*n* (proportion of users)	Reasons for discontinuing mitotane	*n*
Any adverse effect	31 (91%)	Any reason	11
Any gastrointestinal symptom	21 (62%)	Increased liver enzymes	3
Nausea	13 (38%)	Nausea and neurocognitive symptoms	3
Diarrhoea	7 (21%)	Diarrhoea	2
Loss of appetite	5 (15%)	Abdominal pain	1
Abdominal pain	1 (3%)	Back pain	1
Tiredness/fatigue	5 (15%)	Leukopenia	1
Vertigo	6 (18%)		
Neurocognitive symptoms	7 (21%)		

Ten patients received other systemic treatments, including etoposide-doxorubicin-cisplatin (EDP), carboplatin and streptozocin. Two patients achived complete response with sustained remission of > 5 years (one (ENSAT II) treated with mitotane and carboplatin, and one (ENSAT III) with mitotane and EDP combination therapy). Five patients received palliative radioherapy for metastases.

### Survival

**Fig. 2:**
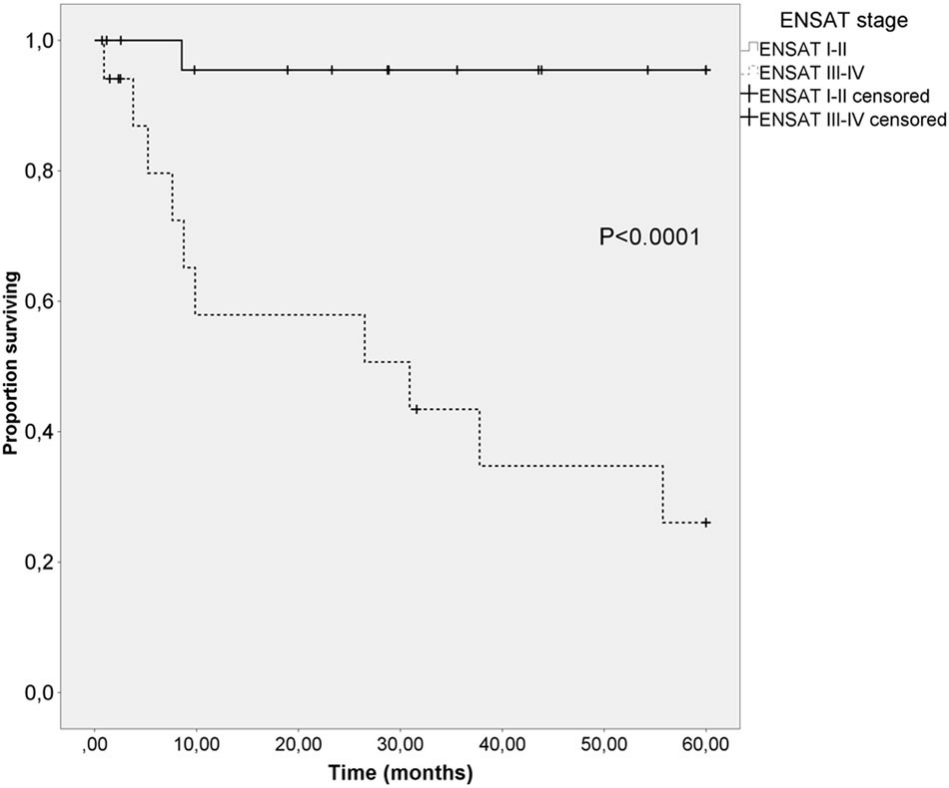
Comparison of overall survival rates of ENSAT stage I–II and III–IV patients using Kaplan–Meier method

Five-year overall survival was 67% and, for ENSAT I, II, III and IV 100%, 93%, 60% and 11% respectively. The difference between ENSAT I–II vs. III–IV was 96% vs. 26%, respectively (*p* < 0.0001; Fig. 2). Median follow-up was 35 months, 14/43 (33%) patients died during follow-up (0/8 stage I, 3/17 (18%) stage II, 3/8 (38%) stage III and 8/9 (89%) stage IV). Risk of death was higher in ENSAT stage III–IV compared to I–II (hazard ratio 9.29, 95% CI 2.56–33.66) (*p* = 0.001). A Ki-67 > 19% associated with poorer survival (hazard ratio 4.4; 95% CI 1.3–14.3) (*p* = 0.02). Helsinki score ( ≤ 17 vs. > 17) did not predict survival (*p* = 0.22), neither did age ( ≤ 55 vs. > 55 years; *p* = 0.96). Symptomatic or incidentally discovered disease (*p* = 0.43), tumour size (*p* = 0.15), hormonal hypersecretion (*p* = 0.54) or gender (*p* = 0.14) did not predict survival. Open surgery tended to associate with poorer survival than laparoscopic surgery (hazard ratio 4.34, 95% CI 0.94–20.12) (*p* = 0.06), this tendency disappeared after adjustment for ENSAT stage. Those who did not receive mitotane had poorer survival compared to those who did (hazard ratio 5.24, 95% CI 1.42–19.38) (*p* = 0.01).

## Discussion

We report on a modern series of ACC, including all patients diagnosed at a single centre in years 2002–2018 and demonstrate that almost 80% nowadays present as asymptomatic incidentally discovered adrenal masses, and that on nonenhanced CT, HU was > 20 for all cases. This is in contrast to the literature, reporting incidental tumour finding in 10–15% of ACC, probably reflecting historical cases diagnosed in 1970–1990 [2, 20, 21]. Adrenal incidentalomas are common due to frequent imaging, the majority representing nonfunctioning adrenal adenomas for which neither surgery nor further imaging is needed [22]. Most adrenal adenomas are homogenous, < 4 cm large and lipid rich, and thus characterised by < 10 HU on nonenhanced CT [22, 28]. ACC and pheochromocytoma are the most important differential diagnoses and must be adequately diagnosed. A German study concluded that HU > 21 provides the best accuracy for diagnosing ACC (96% sensitivity, 80% specificity), however, Weiss scores were not reported [29]. Radiological features of early, small ACCs are rarely reported in the literature [30] and may easily be overlooked. The smallest ACCs of 2 and 2.5 cm in the present study were incidental findings on chest and trauma CT (a 59-year old woman, a 22-year old male), with noncontrast HUs of 34 and 40, and Weiss scores of 7 and 4, respectively. Ozsari et al. [31] reported substantial diagnostic delay of up to 89 months in ACC, in a series of patients with incidentally found adrenal tumours characterised by a high noncontrast HU and small ( < 4 cm) tumour size. The authors concluded that presumed benign nature of pre-existing masses based on size is the main reason for delayed ACC diagnosis.

Data on the incidence of ACC is scarce. We assessed nationwide incidence in 2001–2015 and it is close to the previously reported 1 per million persons annually. A Dutch study reported a declining incidence trend [1], while a recent report from the SEER database, U.S.A, reported increasing trends (597 cases in 1995–2004 compared to 933 cases in 2005–2014) [32].

Open surgery is considered the standard approach for large tumours with high suspicion of or confirmed ACC, at least for tumours demonstrating local invasion. The recent ESE Clinical Practice Guidelines on ACC [33] suggest laparoscopic surgery for tumours < 6 cm without evidence of invasion. In the present study, four adult patients with tumour diameter 6–10 cm, not characterised by invasion successfully underwent laparoscopic surgery. Our results indicate that laparoscopic surgery can be applied in selected cases also for tumours > 6 cm. Importantly, laparoscopic surgery enabled significantly shorter hospitalisation compared to open surgery, and did not impair survival. Two cases were converted because the tumour obstructed the view and one because of adherence to the pancreas. Surgery for ACC, especially laparoscopic, must be reserved for high-volume centres [34, 35]. In 2016 and 2017, the number of laparoscopic adrenalectomies performed at the Helsinki University Hospital was 39 and 51, respectively. In advanced ACC, survival is still very poor, and metastatic ACC is characterised by higher mutation rate and tumour heterogeneity than the primary tumours [36]. As medical treatment options are limited, surgery of metastases should be considered in selected cases. In the present study, four patients underwent resection of metastases, and three clearly benefited, two (ENSAT I and IV; lung metastases, lung metastasis followed by liver metastasis) are in remission (survival > 6 and > 9 years), the third patient (ENSAT II; recurring lung metastases) is alive with prolonged survival of > 10 years.

Of ten patients undergoing further systemic therapy, one treated with mitotane and carboplatin and one with mitotane and EDP (combination therapy, ENSAT stage II and III, respectively) achieved complete response and are alive ( > 5 years).

In the present cohort, 5-year survival rates were 100%, 93%, 60% for ENSAT I, II and III, respectively. This compares favourably to older cohorts [10, 37–40] and probably reflects the high percentage of patients undergoing surgery (88%) and mitotane therapy (79%). However, for ENSAT stage IV, 5-year survival remains poor (11%). ENSAT stage and Ki67 > 19% predicted survival and, as previously reported, mitotane therapy was associated with better survival [7, 33]. Berruti et al. [41] demonstrated that overt clinical hypercortisolism was a negative prognostic factor in ACC. In the present study, 41% had biochemically verified hypercortisolism and this did not predict outcome. The study by Berruti et al. was much larger than the present one, in addition, hypercortisolism was defined differently. Currently, the impact of cortisol secretion on survival in ACC remains uncertain [2].

Mitotane frequently causes side-effects and these were quite common (91%) also in the present study. Mitotane therapy associates with better survival, it is thus important to tailor the treatment according to best practice, including drug concentration monitoring and adequate replacement with rather high hydrocortisone doses, as well as thyroxine and testosterone [42].

As ACC is a very rare cancer, the sample size of the present study was small and this is a clear limitation. Larger series are needed to confirm some of the findings of the present study, such as percentage of cases presenting as incidental cases in other modern series, better characteristics of small ACCs, the role of laparoscopic surgery and surgery of metastases. It is unclear why most of the ACCs were found in the left and not the right adrenal.

In conclusion, contemporary ACC predominantly presents as an incidental imaging finding, characterised by HU > 20 on nonenhanced CT but variable tumour size (20–196 mm). Malignancy cannot be ruled out by small tumour size only. The 5-year survival of 96% in ENSAT stage I–III compares favourably to previous studies.

## References

[1] Kerkhofs TM, Verhoeven RH, Van der Zwan JM, Dieleman J, Kerstens MN, Links TP, Van de Poll-Franse LV, Haak HR (2013). Adrenocortical carcinoma: a population-based study on incidence and survival in the Netherlands since 1993. Eur. J. Cancer.

[2] Jouinot A, Bertherat J (2018). Management of Endocrine Disease Adrenocortical carcinoma: differentiating the good from the poor prognosis tumors. Eur. J. Endocrinol..

[3] Fassnacht M, Johanssen S, Quinkler M, Bucsky P, Willenberg HS, Beuschlein F, Terzolo M, Mueller HH, Hahner S, Allolio B, German Adrenocortical Carcinoma Registry Group, European Network for the Study of Adrenal Tumors (2009). Limited prognostic value of the 2004 International Union Against Cancer staging classification for adrenocortical carcinoma: proposal for a Revised TNM Classification. Cancer.

[4] Assie G, Antoni G, Tissier F, Caillou B, Abiven G, Gicquel C, Leboulleux S, Travagli JP, Dromain C, Bertagna X, Bertherat J, Schlumberger M, Baudin E (2007). Prognostic parameters of metastatic adrenocortical carcinoma. J. Clin. Endocrinol. Metab..

[5] Kebebew E, Reiff E, Duh QY, Clark OH, McMillan A (2006). Extent of disease at presentation and outcome for adrenocortical carcinoma: have we made progress?. World J. Surg..

[6] Bilimoria KY, Shen WT, Elaraj D, Bentrem DJ, Winchester DJ, Kebebew E, Sturgeon C (2008). Adrenocortical carcinoma in the United States: treatment utilization and prognostic factors. Cancer.

[7] Else T, Williams AR, Sabolch A, Jolly S, Miller BS, Hammer GD (2014). Adjuvant therapies and patient and tumor characteristics associated with survival of adult patients with adrenocortical carcinoma. J. Clin. Endocrinol. Metab..

[8] Else T, Kim AC, Sabolch A, Raymond VM, Kandathil A, Caoili EM, Jolly S, Miller BS, Giordano TJ, Hammer GD (2014). Adrenocortical carcinoma. Endocr. Rev..

[9] Ayala-Ramirez M, Jasim S, Feng L, Ejaz S, Deniz F, Busaidy N, Waguespack SG, Naing A, Sircar K, Wood CG, Pagliaro L, Jimenez C, Vassilopoulou-Sellin R, Habra MA (2013). Adrenocortical carcinoma: clinical outcomes and prognosis of 330 patients at a tertiary care center. Eur. J. Endocrinol..

[10] Icard P, Goudet P, Charpenay C, Andreassian B, Carnaille B, Chapuis Y, Cougard P, Henry JF, Proye C (2001). Adrenocortical carcinomas: surgical trends and results of a 253-patient series from the French Association of Endocrine Surgeons study group. World J. Surg..

[11] Wang S, Chen SS, Gao WC, Bai L, Luo L, Zheng XG, Luo Y (2017). Prognostic factors of adrenocortical carcinoma: An analysis of the surveillance epidemiology and end results (SEER) Database. Asian Pac. J. Cancer. Prev..

[12] Michalkiewicz E, Sandrini R, Figueiredo B, Miranda EC, Caran E, Oliveira-Filho AG, Marques R, Pianovski MA, Lacerda L, Cristofani LM, Jenkins J, Rodriguez-Galindo C, Ribeiro RC (2004). Clinical and outcome characteristics of children with adrenocortical tumors: a report from the International Pediatric Adrenocortical Tumor Registry. J. Clin. Oncol..

[13] Pennanen M, Heiskanen I, Sane T, Remes S, Mustonen H, Haglund C, Arola J (2015). Helsinki score-a novel model for prediction of metastases in adrenocortical carcinomas. Hum. Pathol..

[14] Beuschlein F, Weigel J, Saeger W, Kroiss M, Wild V, Daffara F, Libe R, Ardito A, Al Ghuzlan A, Quinkler M, Osswald A, Ronchi CL, de Krijger R, Feelders RA, Waldmann J, Willenberg HS, Deutschbein T, Stell A, Reincke M, Papotti M, Baudin E, Tissier F, Haak HR, Loli P, Terzolo M, Allolio B, Muller HH, Fassnacht M (2015). Major prognostic role of Ki67 in localized adrenocortical carcinoma after complete resection. J. Clin. Endocrinol. Metab..

[15] Tran TB, Postlewait LM, Maithel SK, Prescott JD, Wang TS, Glenn J, Phay JE, Keplinger K, Fields RC, Jin LX, Weber SM, Salem A, Sicklick JK, Gad S, Yopp AC, Mansour JC, Duh QY, Seiser N, Solorzano CC, Kiernan CM, Votanopoulos KI, Levine EA, Hatzaras I, Shenoy R, Pawlik TM, Norton JA, Poultsides GA (2016). Actual 10-year survivors following resection of adrenocortical carcinoma. J. Surg. Oncol..

[16] Terzolo M, Angeli A, Fassnacht M, Daffara F, Tauchmanova L, Conton PA, Rossetto R, Buci L, Sperone P, Grossrubatscher E, Reimondo G, Bollito E, Papotti M, Saeger W, Hahner S, Koschker AC, Arvat E, Ambrosi B, Loli P, Lombardi G, Mannelli M, Bruzzi P, Mantero F, Allolio B, Dogliotti L, Berruti A (2007). Adjuvant mitotane treatment for adrenocortical carcinoma. N. Engl. J. Med..

[17] Berruti A, Grisanti S, Pulzer A, Claps M, Daffara F, Loli P, Mannelli M, Boscaro M, Arvat E, Tiberio G, Hahner S, Zaggia B, Porpiglia F, Volante M, Fassnacht M, Terzolo M (2017). Long-term outcomes of adjuvant mitotane therapy in patients with radically resected adrenocortical carcinoma. J. Clin. Endocrinol. Metab..

[18] Fassnacht M, Terzolo M, Allolio B, Baudin E, Haak H, Berruti A, Welin S, Schade-Brittinger C, Lacroix A, Jarzab B, Sorbye H, Torpy DJ, Stepan V, Schteingart DE, Arlt W, Kroiss M, Leboulleux S, Sperone P, Sundin A, Hermsen I, Hahner S, Willenberg HS, Tabarin A, Quinkler M, de la Fouchardiere C, Schlumberger M, Mantero F, Weismann D, Beuschlein F, Gelderblom H, Wilmink H, Sender M, Edgerly M, Kenn W, Fojo T, Muller HH, Skogseid B, FIRM-ACT Study Group (2012). Combination chemotherapy in advanced adrenocortical carcinoma. N. Engl. J. Med..

[19] Megerle F, Herrmann W, Schloetelburg W, Ronchi CL, Pulzer A, Quinkler M, Beuschlein F, Hahner S, Kroiss M, Fassnacht M, German ACC Study Group (2018). Mitotane monotherapy in patients with advanced adrenocortical carcinoma. J. Clin. Endocrinol. Metab..

[20] Dackiw AP, Lee JE, Gagel RF, Evans DB (2001). Adrenal cortical carcinoma. World J. Surg..

[21] Johanssen S, Hahner S, Saeger W, Quinkler M, Beuschlein F, Dralle H, Haaf M, Kroiss M, Jurowich C, Langer P, Oelkers W, Spahn M, Willenberg HS, Mader U, Allolio B, Fassnacht M (2010). Deficits in the management of patients with adrenocortical carcinoma in Germany. Dtsch. Arztebl Int..

[22] Fassnacht M, Arlt W, Bancos I, Dralle H, Newell-Price J, Sahdev A, Tabarin A, Terzolo M, Tsagarakis S, Dekkers OM (2016). Management of adrenal incidentalomas: European Society of Endocrinology Clinical Practice Guideline in collaboration with the European Network for the Study of Adrenal Tumors. Eur. J. Endocrinol..

[23] Sane T, Schalin-Jantti C, Raade M (2012). Is biochemical screening for pheochromocytoma in adrenal incidentalomas expressing low unenhanced attenuation on computed tomography necessary?. J. Clin. Endocrinol. Metab..

[24] L.M. Weiss, L.J. Medeiros, A.L. Vickery, Jr., Pathologic features of prognostic significance in adrenocortical carcinoma. Am. J. Surg. Pathol. **13**, 202–206 (1989). 10.1097/00000478-198903000-00004 2919718

[25] Weiss LM (1984). Comparative histologic study of 43 metastasizing and nonmetastasizing adrenocortical tumors. Am. J. Surg. Pathol..

[26] Dindo D, Demartines N, Clavien PA (2004). Classification of surgical complications: a new proposal with evaluation in a cohort of 6336 patients and results of a survey. Ann. Surg..

[27] Fassnacht M, Allolio B (2009). Clinical management of adrenocortical carcinoma. Best Pract. Res. Clin. Endocrinol. Metab..

[28] Schalin-Jantti C, Raade M, Hamalainen E, Sane T (2015). A 5-year prospective follow-up study of lipid-rich adrenal incidentalomas: No tumor growth or development of hormonal hypersecretion. Endocrinol. Metab. (Seoul).

[29] Petersenn S, Richter PA, Broemel T, Ritter CO, Deutschbein T, Beil FU, Allolio B, Fassnacht M, German ACC Study Group (2015). Computed tomography criteria for discrimination of adrenal adenomas and adrenocortical carcinomas: analysis of the German ACC registry. Eur. J. Endocrinol..

[30] Barnett CC, Varma DG, El-Naggar AK, Dackiw AP, Porter GA, Pearson AS, Kudelka AP, Gagel RF, Evans DB, Lee JE (2000). Limitations of size as a criterion in the evaluation of adrenal tumors. Surgery.

[31] Ozsari L, Kutahyalioglu M, Elsayes KM, Vicens RA, Sircar K, Jazaerly T, Waguespack SG, Busaidy NL, Cabanillas ME, Dadu R, Hu MI, Vassilopoulou-Sellin R, Jimenez C, Lee JE, Habra MA (2016). Preexisting adrenal masses in patients with adrenocortical carcinoma: clinical and radiological factors contributing to delayed diagnosis. Endocrine.

[32] Sharma E, Dahal S, Sharma P, Bhandari A, Gupta V, Amgai B, Dahal S (2018). The characteristics and trends in adrenocortical carcinoma: A United States population based study. J. Clin. Med. Res..

[33] Fassnacht M, Dekkers O, Else T, Baudin E, Berruti A, de Krijger RR, Haak HR, Mihai R, Assie G, Terzolo M (2018). European Society of Endocrinology Clinical Practice Guidelines on the Management of Adrenocortical Carcinoma in Adults, in collaboration with the European Network for the Study of Adrenal Tumors. Eur. J. Endocrinol..

[34] Barczynski M, Konturek A, Golkowski F, Cichon S, Huszno B, Peitgen K, Walz MK (2007). Posterior retroperitoneoscopic adrenalectomy: a comparison between the initial experience in the invention phase and introductory phase of the new surgical technique. World J. Surg..

[35] Schreinemakers JM, Kiela GJ, Valk GD, Vriens MR, Rinkes IH (2010). Retroperitoneal endoscopic adrenalectomy is safe and effective. Br. J. Surg..

[36] Gara SK, Lack J, Zhang L, Harris E, Cam M, Kebebew E (2018). Metastatic adrenocortical carcinoma displays higher mutation rate and tumor heterogeneity than primary tumors. Nat. Commun..

[37] Asare EA, Wang TS, Winchester DP, Mallin K, Kebebew E, Sturgeon C (2014). A novel staging system for adrenocortical carcinoma better predicts survival in patients with stage I/II disease. Surgery.

[38] Kerkhofs TM, Ettaieb MH, Hermsen IG, Haak HR (2015). Developing treatment for adrenocortical carcinoma. Endocr. Relat. Cancer.

[39] Libe R, Borget I, Ronchi CL, Zaggia B, Kroiss M, Kerkhofs T, Bertherat J, Volante M, Quinkler M, Chabre O, Bala M, Tabarin A, Beuschlein F, Vezzosi D, Deutschbein T, Borson-Chazot F, Hermsen I, Stell A, Fottner C, Leboulleux S, Hahner S, Mannelli M, Berruti A, Haak H, Terzolo M, Fassnacht M, Baudin E (2015). Prognostic factors in stage III-IV adrenocortical carcinomas (ACC): an European Network for the Study of Adrenal Tumor (ENSAT) study. Ann. Oncol..

[40] Fassnacht M, Johanssen S, Fenske W, Weismann D, Agha A, Beuschlein F, Fuhrer D, Jurowich C, Quinkler M, Petersenn S, Spahn M, Hahner S, Allolio B, German ACC Registry Group (2010). Improved survival in patients with stage II adrenocortical carcinoma followed up prospectively by specialized centers. J. Clin. Endocrinol. Metab..

[41] Berruti A, Fassnacht M, Haak H, Else T, Baudin E, Sperone P, Kroiss M, Kerkhofs T, Williams AR, Ardito A, Leboulleux S, Volante M, Deutschbein T, Feelders R, Ronchi C, Grisanti S, Gelderblom H, Porpiglia F, Papotti M, Hammer GD, Allolio B, Terzolo M (2014). Prognostic role of overt hypercortisolism in completely operated patients with adrenocortical cancer. Eur. Urol..

[42] Terzolo M, Ardito A, Zaggia B, Laino F, Germano A, De Francia S, Daffara F, Berruti A (2012). Management of adjuvant mitotane therapy following resection of adrenal cancer. Endocrine.

